# GLIM Criteria for Malnutrition in Surgical IBD Patients: A Pilot Study

**DOI:** 10.3390/nu12082222

**Published:** 2020-07-25

**Authors:** Camilla Fiorindi, Cristina Luceri, Gabriele Dragoni, Guya Piemonte, Stefano Scaringi, Fabio Staderini, Anita Nannoni, Ferdinando Ficari, Francesco Giudici

**Affiliations:** 1 Department of Health Science, University of Florence, 50139 Florence, Italy; camilla.fiorindi@unifi.it (C.F.); guya.piemonte@gmail.com (G.P.); nannonia@aou-careggi.toscana.it (A.N.); 2 NEUROFARBA Department, Pharmacology and Toxicology Section, University of Florence, 50139 Florence, Italy; cristina.luceri@unifi.it; 3 Department of Experimental and Clinical Biomedical Sciences “Mario Serio”, University of Florence, 50139 Florence, Italy; gabriele.dragoni@unifi.it; 4 Department of Experimental and Clinical Medicine, University of Florence, 50139 Florence, Italy; stefano.scaringi@unifi.it (S.S.); fabio.staderini@unifi.it (F.S.); ferdinando.ficari@unifi.it (F.F.)

**Keywords:** GLIM, IBD, surgery, nutrition, screening, malnutrition, body composition, sarcopenia, risk

## Abstract

Background: A gold standard method for malnutrition diagnosis is still lacking in Inflammatory Bowel Disease (IBD). Objective: The aims of this study are to determine the prevalence of malnutrition in IBD patients according with recently published Global Leadership Initiative on Malnutrition (GLIM) criteria, to detect the factors contributing to the onset of malnutrition, and to evaluate the most accurate predictor of malnutrition risk within the available nutritional screening tools. Methods: Fifty-three consecutive adult IBD patients [38 Crohn’s disease (CD) and 15 ulcerative colitis (UC)] had been assessed preoperatively by a multidisciplinary IBD team before undergoing elective surgery. Several malnutrition risk tools were tested, such as NRS-2002, MUST, MST, MIRT, and SaskIBD-NR. The statistical association of independent GLIM variables with baseline characteristics of patients was explored as well as the concordance with the European Society for Clinical Nutrition and Metabolism (ESPEN 2015) and the screening tools. Results: Twenty-two IBD patients (42%) were malnourished according to GLIM criteria, of which 13 were CD (34%) and 9 UC (60%). The etiological criteria of inflammation and reduction of food intake were present in 51% and 19% of our patients, respectively. The prevalence of GLIM phenotypic criteria was 28%, 28% and 34% for BMI, Free Fat Mass Index (FFMI) and unintended weight loss (UWL), respectively. The presence of ileostomy was statistically associated with a higher prevalence of BMI (*p* = 0.030), FFMI (*p* = 0.030) and UWL (*p* = 0.002) values lower than the GLIM criteria cut-offs, while secondary surgery is associated with a decrease in FFMI (*p* = 0.017) and UWL (*p* = 0.041). The sensitivity of the tested nutritional screening tools, compared with the GLIM prevalence of malnutrition, was not satisfactory (between 50 and 82%). Conclusions: GLIM has a higher rate of malnutrition detection than ESPEN 2015, as malnutrition in IBD seems linked to inflammation and secondary malabsorption even without a reduction of food intake. The sensitivity of the screening tools is lower than the specificity when compared with GLIM criteria for malnutrition diagnosis.

## 1. Introduction

Malnutrition is common in patients with inflammatory bowel diseases (IBD) and often leads to impaired body composition with loss of body mass lean and nutritional deficiencies. Its etiology is multifactorial depending on the combination of various factors, such as the inflammatory response, the clinical complications of the disease (strictures, abscesses and fistulas), and the previous surgical resections. All these features are responsible for malabsorption and nutrient loss [1,2]. This scenario often occurs in Crohn’s disease (CD) but may also be present in chronically active and severe forms of ulcerative colitis (UC) [3,4,5,6,7]. According to published evidence, malnutrition affects a large portion of IBD patients, ranging from 20 to 70% depending on the adopted parameters of nutritional assessment [8,9,10,11].

In IBD patients, body composition is mainly affected by gender, therapies and disease specific features (duration, activity and localization) [6,12]. The European Society for Clinical Nutrition and Metabolism (ESPEN) guidelines on clinical nutrition in IBD emphasize the increased risk of malnutrition, also including normal and overweight subjects whose lean mass deficiency can be masked when using simple anthropometric measurements [13]. Unfortunately, different parameters have been considered in previous works to assess the preoperative nutritional risk, suggesting that a gold standard method for malnutrition diagnosis is still lacking, especially in IBD [14,15]. Recently, the Global Leadership Initiative on Malnutrition (GLIM) gathered together the major clinical nutrition societies to reach a global consensus on the identification of criteria for the diagnosis of malnutrition in clinical settings [16].

To our knowledge, no data have been published so far on the prospective adoption of this new approach in the IBD population. The aim of our study was to determine the prevalence of malnutrition in IBD patients according with recently published GLIM criteria and to evaluate these criteria as a plausible gold standard method for malnutrition diagnosis in this setting. A comprehensive analysis of existing indicators used for screening and assessment of malnutrition has been conducted to identify criteria worthy of consideration. Secondary endpoints were to detect the factors contributing to the onset of malnutrition and to determine the best predictive test of GLIM-diagnosed malnutrition among the currently available nutritional screening tools in IBD.

## 2. Methods

### 2.1. Study Population

The present study was prospectively conducted at Careggi University Hospital in Florence (Italy) between December 2018 and November 2019, after obtaining approval by the Local Ethical Scientific Committee (12382_BIO) and informed consent from each patient. The study population included consecutive adult patients with a scheduled elective surgery for CD or UC. All cases had been assessed preoperatively by a multidisciplinary IBD team (composed of dedicated surgeons, gastroenterologists, radiologists and dietitians).

### 2.2. Study Design

The nutritional status of each patient was evaluated by a dedicated dietitian from our IBD Unit during the pre-hospitalization assessment. All necessary data were collected as follows: anthropometric parameters (body weight, height, body mass index (BMI), unintended weight loss (UWL)); food and nutrition related history; body composition through bio-impedance vector analysis (BIVA); biochemical markers of inflammation (C-reactive protein (CRP), white blood cells (WBC) count, fibrinogen) gastrointestinal symptoms (diarrhea, nausea, vomiting, bloating/abdominal pain and decreased appetite). The Duke activity status index (DASI) was adopted to evaluate the functional capacity of patients. According to GLIM criteria, the diagnosis of malnutrition was based on the presence of at least one phenotypic criterion (UWL; low BMI; reduced free fat mass (FFM)) and at least one etiologic criterion (reduced food intake or assimilation; inflammation). The results were analysed to establish the severity of malnutrition, separated into Stage 1 (moderate) and Stage 2 (severe) [16]. The results were compared with ESPEN 2015 criteria for diagnosis of malnutrition [17] to evaluate their concordance. All patients were screened for malnutrition with different existing nutritional tools such as the Nutritional Risk Screening (NRS-2002) [18], the Malnutrition Universal Screening Tool (MUST) [19], the Malnutrition Screening Tool (MST) [20] and with two other tests specifically designed for the IBD population, i.e., the Malnutrition Inflammation Risk Tool (MIRT) [21] and the Saskatchewan IBD–Nutrition Risk (SaskIBD-NR) [22]. Stool type was reported according to the Bristol stool chart [23].

Table 1 shows GLIM parameters included in the different screening tools and the cut-off that was set for every screening tool to diagnose a high nutritional risk. The prevalence of high nutritional risk obtained with these different tests was related to the diagnosis of malnutrition obtained with the GLIM criteria to investigate their relationship.

### 2.3. GLIM Criteria

GLIM criteria are based on:

1. BMI: calculated as weight (kg) divided by squared height (meters). BMI cut-offs for malnutrition risk are <20 kg/m^2^ if < 70 years, and <22 kg/m^2^ if >70 years.
2. UWL: non volitional weight loss >5% within the last 6 months, or >10% beyond the last 6 months.
3. Free Fat Mass Index (FFMI): calculated as FFM (kg) divided by squared height (meters). FFMI cut-offs for malnutrition risk are <17 kg/m^2^ for men and <15 kg/m^2^ for women.
4. Food and nutrition related history: according to GLIM criteria for malnutrition, reduction of >50% of Energy Requirement (ER) >1 week, or any reduction for >2 weeks is considered at risk.
5. State of inflammation: Chronic or recurrent mild-to-moderate inflammation is likely to be associated with malignant disease, chronic obstructive pulmonary disease, congestive heart failure, chronic renal disease or any disease which is chronic or recurrent.

### 2.4. Statistical Analysis

Data are presented as mean ± SD and percentages, as appropriate. Categorical variables were analysed using Fisher’s exact test and continuous variables with Student’s t-test, with a statistically significant association set at *p* < 0.05. The agreement between the different criteria used for the diagnosis of malnutrition (GLIM criteria vs. ESPEN 2015 criteria) and between the five screening tools was calculated with Cohen’s kappa coefficient (K). Sensitivity, specificity, likelihood ratio (LR) and area under the receiver operating characteristic (ROC) curve have been calculated for each test to evaluate the reliability of the several malnutrition screening tools compared to malnutrition diagnosis according to GLIM criteria.

## 3. Results

A total of 53 IBD patients were included, 38 CD (72%) and 15 UC (28%). Table 2 summarizes the baseline characteristics of the patients. The mean duration of disease was 11 years and was similar between the two groups. Patients at first operation were 53%, while 47% have had previous abdominal surgery: in fact, fourteen CD patients (37%) were scheduled for surgery due to a surgical recurrence, whereas 11 UC patients (74%) had to complete the treatment of total proctocolectomy with ileal-pouch anal anastomosis (IPAA). In particular, 5 UC patients (33%) were scheduled for total colectomy with ileostomy and rectal stump (first time surgery for acute severe UC), eight patients (53%) for proctectomy, IPAA and loop ileostomy (second time surgery for acute severe UC) and two patients (13%) for total proctocolectomy with IPAA and loop ileostomy. Regarding CD patients, 81% had isolated ileal disease and 69% had stricturing behaviour. Globally, 11 IBD patients had an ileostomy, of whom 10 UC (67% of UC group) and one CD (3% of CD group).

Twelve UC patients (80%) reported Bristol type 5–6 stool (diarrhea), while the remaining three (20%) had normal stool (type 4). Eighteen CD patients (48%) reported Bristol type 5–6 stool (including the only patient with stoma), 18 patients (47%) had normal stool (type 3–4) and only two (5%) described type 2 stool. More than three gastrointestinal (GI) symptoms were reported by 11 CD patients (29%) only. The other patients (27 CD and 15 UC) had three or less GI symptoms.

A low BMI was reported in 24% of CD and 40% of UC patients. Three patients were obese (one UC and two CD) and seven were overweight (one UC and six CD). None of them had sarcopenia. The most prevalent phenotypic criterion was the UWL (34%). In particular, UWL occurred more in UC than in CD (*p* = 0.0224). Reduced lean body mass, according to FFMI and measured with BIVA, was present in 33% of CD and 30% of UC male patients, respectively, and in 12% of CD and 60% of UC female patients, respectively (*p* = 0.054). Only 20% of patients had reduced oral intake, of whom only one was below 50% of the total energy requirement. Many patients had eliminated some food groups (fiber-rich foods, lactose-containing foods) due to associated symptoms (diarrhea, vomiting, bloating/abdominal pain) or well-known intestinal strictures.

With regard to the inflammation state, CRP was > 9 mg/L in 59% of CD patients and in 40% of UC patients. WBC count was significantly above normal limit (10 × 10^9^/L) in CD (46%) compared to UC (13%) (*p* = 0.0312) (Table 3).

The prevalence of malnutrition according to GLIM criteria was 42% (15% stage 1, and 27% stage 2). In total, 13 CD patients (34%) and 9 UC patients (60%) were malnourished. The mean age and disease duration were lower in the malnourished group, although not statistically significant. There was an equal distribution of gender, smoking habit and DASI between the two groups. The malnourished CD patients showed a prevalence of fistulizing disease behaviour when compared with the well-nourished group (*p* = 0.0199) (Table 4). The concordance between GLIM diagnosis and ESPEN 2015 diagnosis was moderate-good (k = 0.672). The 14 malnourished patients according to ESPEN 2015 were the same 14 patients that had stage 2 malnutrition according to GLIM criteria (Table 5).

The statistical association of independent GLIM variables with baseline characteristics (age, sex, duration of disease, previous surgery, presence of stoma) of patients has been carried out. The analysis has showed that patients with previous surgery had lower FFMI and UWL than patients at their first surgery (*p* = 0.017 and *p* = 0.041 for FFMI and UWL, respectively), thus remaining below the risk cut-offs adopted by GLIM. Similarly, the presence of ileostomy was associated with a higher prevalence of lower BMI, FFMI and UWL than GLIM criteria (BMI *p* = 0.030; FFMI *p* = 0.030; UWL *p* = 0.002). No statistical association was found on analyzing baseline characteristics and GLIM etiological criteria. No statistical association was found between GLIM malnutrition diagnosis and medical therapy (mesalamine, steroids, thiopurines, biological drugs) continuously assumed during the three months before surgery.

### 3.1. Concordance between the Different Screening Tools

The prevalence of high nutritional risk was different depending on the screening test adopted (Table 6). The tests that identified the highest percentage of malnutrition risk were the NRS-2002 (40%) and the MIRT (40%), whereas the one that detected the lowest was the SaskIBD-NR (25%). The calculated prevalence of high nutritional risk according to the different nutritional screening tools was lower than the malnutrition assessed with GLIM criteria (42%).

**Figure 1:**
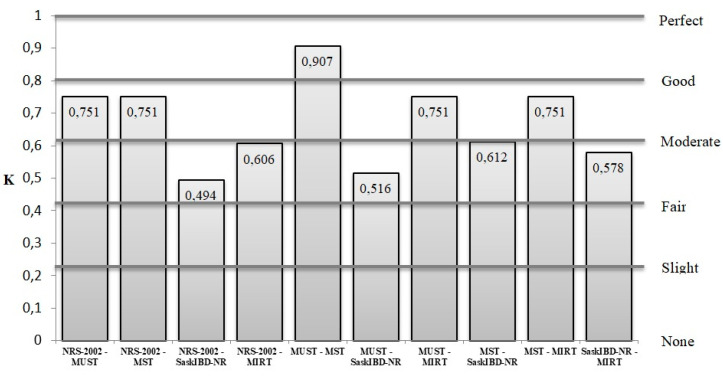
Concordance (K) between the nutritional screening tools.

The screening tools with excellent concordance were MUST and MST (k = 0.907), followed by NRS-2002 and MUST (k = 0.751), NRS-2002 and MST (k = 0.751), MIRT and MUST (k = 0.751), MIRT and MST (k = 0.751), and SaskIBD-NR and MST (k = 0.612). The other comparisons showed only a moderate agreement (k < 0.6) (Figure 1).

### 3.2. Concordance between GLIM Diagnosis of Malnutrition and Nutritional Screening Tools

Considering the GLIM diagnosis of malnutrition, the nutritional screening tools with fewer false negatives were the NRS-2002 and the MIRT (*n* = 4), while the one with the most was the SaskIBD-NR (*n* = 11). The MUST and MST showed eight false negatives. Associating GLIM diagnosis of malnutrition with the different screening tools adopted, we found LR values > 1 (the higher the value, the higher the association). Area under the ROC curve was also calculated to predict the validity of the different tools (Table 7).

## 4. Discussion

Several studies have showed that IBD patients scheduled for surgery are often malnourished, thus increasing the incidence of postoperative complications [24]. For this reason, it is important to have an accurate method of diagnosis and assessment of malnutrition in IBD patients. According to the recent GLIM criteria, we found a prevalence of malnutrition in 42% of our surgical IBD population. When comparing our data with the few available studies, it appears that a higher percentage of malnourished patients is detected using GLIM criteria. In fact, a multi-center, observational, prospective study conducted in 30 Spanish centers (333 IBD patients) reported an overall prevalence of malnutrition at around 16%, with similar distribution in CD and UC patients [11]. According to Pulley et al., mild to moderate malnutrition was detected in 17 IBD patients (16%) out of a cohort of 107 IBD cases, with concomitant active flare in only seven patients [25]. Another study reported a prevalence of malnutrition in 6% of CD and 7% of UC inpatients, but protein-calorie malnutrition was retrospectively identified by ICD-9-CM diagnostic codes only [5]. These data could relate to the different cohort of IBD patients analyzed, as our patients were affected by active disease requiring surgery, thus explaining the higher percentage of malnutrition. Furthermore, it is still difficult to correlate our data with those already reported in the literature, because a gold-standard approach to define malnutrition in IBD patients has not yet been identified [24]. Mijac et al. reported that the adoption of several parameters of malnutrition in 76 acute IBD patients (53 UC, 23 CD) resulted in a prevalence of undernutrition between 25% and 69.7%, and of severe malnutrition between 1.3% and 31.6%, concluding that a lack of consensus on the exact criteria to define malnutrition in IBD leads to inconsistent and incomparable results [8].

All our patients required surgery due to acute or complicated disease refractory to medical therapy. For this reason, the etiological criterion of inflammation in GLIM influenced the diagnosis of malnutrition, as well as the evaluation of biomarkers, that were increased in 51% of our patients. The other etiological criterion evaluated was the reduction of food intake, reported in 19% of our cohort. This correctly implies that malnutrition can be present and linked to inflammation and secondary malabsorption despite a not significant reduction of the nutritional intake due to an acute or complicated IBD status.

The prevalence of GLIM phenotypic criteria was 28%, 28% and 34% for BMI, FFMI and UWL, respectively. Interestingly, the presence of ileostomy was statistically associated with lower BMI, FFMI and UWL values than GLIM criteria cut-offs, whereas the secondary surgery was associated with a decrease of FFMI and UWL. Therefore, both presence of ileostomy and secondary or recurrent surgery could be included as independent risk factors in nutritional screening tools for IBD patients. Accordingly, sarcopenia, indirectly estimated by FFMI, is associated with an increased risk of postoperative morbidity and mortality and its assessment appears to be necessary before surgery [26,27,28,29].

A separate analysis of UC and CD patients showed a higher prevalence of phenotypic criteria (in particular FFMI and UWL) for UC than for CD patients, whereas the etiological criterion of inflammation appears more prevalent in CD. These latter findings might relate to the fact that most UC patients included in our study had an ileostomy at nutritional evaluation (second time surgery), while CD patients showed a greater inflammatory status related to active disease.

The evaluation of our data in accordance with ESPEN 2015 criteria reported a prevalence of malnutrition at 27%. ESPEN 2015 and GLIM had a moderate-good concordance, but GLIM was able to diagnose more frequently a mild malnutrition thanks to the analysis of the etiological criteria. On the other hand, the two methods showed a 100% concordance for patients with severe malnutrition (stage 2 of GLIM criteria).

Regarding malnutrition screening tools, NRS-2002, MUST, MST as well as IBD-specific tests (MIRT, SaskIBD-NR) are currently the most commonly used in the IBD population [30]. In our cohort, the sensitivity of these nutritional screening tools was not high (50–82%) if compared with GLIM. In fact, the malnutrition rate according to GLIM was constantly superior to the prevalence of high nutritional risk calculated by each screening test. The reason for this could be related to the variable absence of etiological or phenotypical parameters in the different screening tools. Specifically, the NRS-2002 does not include the evaluation of FFMI, the MUST and MST do not include FFMI and inflammation, while the specific tests for IBD include the evaluation of the inflammatory state without considering all phenotypical parameters.

## 5. Conclusions 

In conclusion, this study showed that the factors that most predispose to malnutrition, diagnosed according to GLIM, are the recurrence of the disease/previous IBD surgery and the presence of ileostomy. The sensitivity of screening tools is lower than specificity in relationship to GLIM malnutrition diagnosis. The more accurate is NRS-2002. Furthermore, GLIM has a higher percentage of malnutrition detection than ESPEN 2015, but a question appears legitimate: does it overestimate the nutritional risk, or does it complete the current clinical assessment? As the relationship between malnutrition and postoperative complications in IBD is well known, it is more prudent to overestimate than to underestimate malnutrition to reach a good clinical outcome. Therefore, we believe that after the validation of GLIM a new screening tool including age, weight loss, BMI, reduced food (or nutritional intake or elimination of food groups), inflammation/disease burden, presence of ileostomy and recurrent surgery could be necessary in the assessment of IBD patients requiring surgery.

## Figures and Tables

**Table 1:** Global Leadership Initiative in Malnutrition (GLIM) parameters included in nutritional screening tools used, and nutritional risk cut-off.

	NRS-2002	MUST	MST	SaskIBD-NR	MIRT
Weight loss	Yes	Yes	Yes	Yes	Yes
Low BMI	Yes	Yes	No	No	Yes
Reduced muscle mass	No	No	No	No	No
Reduced food or nutritional intake or Elimination of food groups	Yes	Yes	Yes	Yes	No
Presence of symptoms	No	No	No	Yes	No
Inflammation/disease burden *	Yes	No	No	No	Yes
Age	Yes	No	No	No	No
Score for high nutritional risk	**≥3**	**≥2**	**≥2**	**≥5**	**≥3**

* Acute disease/injury-related: Severe inflammation is likely to be associated with major infection, burns, trauma or closed head injury. Other acute disease/injury-related conditions are likely to be associated with mild-to-moderate inflammation. Chronic disease-related: Severe inflammation is not generally associated with chronic disease conditions. Chronic or recurrent mild-to-moderate inflammation is likely to be associated with malignant disease, chronic obstructive pulmonary disease, congestive heart failure, chronic renal disease or any disease with chronic or recurrent inflammation. NRS-2002 = Nutritional Risk Screening 2002; MUST = Malnutrition Universal Screening Tool; MST = Malnutrition Screening Tool; SaskIBD-NR = Saskatchewan IBD–Nutrition Risk; MIRT = Malnutrition Inflammation Risk Tool; BMI = Body Mass Index.

**Table 2:** Baseline characteristics of Inflammatory Bowel Disease (IBD) patients.

	IBD	CD	UC	p *
Patients, *n* (%)	53	38 (72%)	15 (28%)	
Age, yrs, average, SD	51.08 ± 15.06	52.00 ± 13.73	48.73 ± 18.33	0.48
Median, yrs, (IQR)	54 (41–62)	53.5 (42.2–59.7)	57 (32.5–62.5)	
Males, *n* (%)	31 (58%)	21 (55%)	10 (67%)	0.54
Females, *n* (%)	22 (42%)	17 (45%)	5 (33%)	
Disease duration, yrs (IR)	11 (2.5–17.5)	10 (1.75–17)	12 (3–19)	0.52
First operation, *n* (%)	28 (53%)	24 (63%)	4 (26%)	0.0308
Presence of stoma, *n* (%)	11 (21%)	1 (3%)	10 (67%)	<0.001
Type of stool **, *n* (%)				
Type 2	2 (4%)	2 (5%)	0	>0.999
Type 3	5 (9%)	5 (13%)	0	0.3055
Type 4	16(30%)	13 (34%)	3 (20%)	0.5076
Type 5	23(43%)	14 (37%)	9 (60%)	0.2179
Type 6	7 (13%)	4 (11%)	3 (20%)	0.3894
N° of GI symptoms				
<3, *n* (%)	42 (79%)	27 (71%)	15 (100%)	0.0228
≥3, *n* (%)	11 (21%)	11 (29%)	0	
Crohn’s Disease behaviour				
-stricturing, *n* (%)		25 (69%)		
-fistulizing, *n* (%)		11 (31%)		
Crohn’s Disease’s localization				
-Ileal, *n* (%)		30 (81.1%)		
-Ileocolonic, *n* (%)		3 (8.1%)		
-Colonic, *n* (%)		4 (10.8%)		
UC, *n* (%)				
-Proctitis			7 (46.7%)	
-Left side colitis			1 (6.7%)	
-Extensive colitis			7 (46.7%)	

IQR = interquartile range 25–75%; GI = gastrointestinal; * *p*-values Crohn’s disease (CD) vs. ulcerative colitis (UC); ** According to Bristol stool chart.

**Table 3:** Prevalence of Phenotypic and Etiological criteria in IBD patients.

	CD	UC	p *
BMI, kg/m^2^, mean (Range)	21.7 (20–24)	21.5 (18–25)	0.76
BMI < 20 or 22 kg/m^2^ **, *n* (%)	9 (24%)	6 (40%)	0.313
UWL, *n* (%)	9 (28%)	9 (60%)	0.0224
UWL 5–10% in 6 months, *n* (%)	6 (16%)	5 (33%)	0.1081
UWL ≥ 10% in 6 months, *n* (%)	5 (13%)	4 (27%)	0.1805
FFMI			
-Men, mean, SD	18.7 ± 2.5	17.8 ± 2.1	0.3782
-Men <17 kg/m^2^, *n* (%)	7 (33%)	3 (30%)	> 0.999
-Women, mean, SD	16.3 ± 1.4	15.3 ± 1.3	0.1534
-Women <15 kg/m^2^, *n* (%)	2 (12%)	3 (60%)	0.054
Reduced food intake			
≤50% of ER > 1 week, *n* (%)	1 (3%)	0	>0.999
Any reduction for > 2 weeks, *n* (%)	6 (16%)	3 (20%)	
Inflammation			
CRP > 9 mg/L, *n* (%)	22 (59%)	6 (40%)	0.2327
WGC > 10 × 10^9^/L, *n* (%)	17 (46%)	2 (13%)	0.0312
Fibrinogen > 400 mg/dL, *n* (%)	17 (50%)	5 (36%)	0.5257

* *p*-values CD vs. UC. ** BMI = Body Mass Index (BMI cut-off for age > 70 years); FFMI = Free Fat Mass Index.

**Table 4:** Analysis of continuous and categorical variables in relation to the onset of malnutrition.

	No Malnutrition	Malnutrition	*p*
	According to GLIM	According to GLIM	
Age			
CD, years, mean, SD	52.9 ± 12.9	50.2 ± 15.5	0.5737
UC years, mean, SD	53.5 ± 18.3	45.5 ± 18.7	0.4313
DASI			
CD, points, mean (IR)	50.7 (25.3–58.2)	58.2 (31.3–58.2)	0.4228
UC, points, mean (IR)	45.7 (30.3–52.6)	43.7 (35.9–58.2)	0.6556
Duration of Disease			
CD, years, mean, SD	13.1 ± 12.2	8.6 ± 6.8	0.2304
UC, years, mean, SD	18.0 ± 14.0	10.6 ± 9.6	0.2402
Gender, M			
CD, patients, *n* (%)	15 (71.4%)	6 (28.6%)	0.4232
UC, patients, *n* (%)	5 (50.0%)	5 (50.0%)	
Smokers			
CD, patients, *n* (%)	8 (61.5%)	5 (38.5%)	>0.999
UC	1 (100.0%)	0	
First operation			
CD, patients, *n* (%)	15 (62.5%)	9 (37.5.0%)	>0.999
UC, patients, *n* (%)	3 (75.0%)	1 (25.0%)	
CD behaviour			
Stricturing, patients, *n* (%)	20 (80.0%)	5 (20.0%)	**0.0199**
Fistulizing, patients, *n* (%)	4 (36.4%)	7 (63.6%)	
Disease’s localization			
*CD*			
Small bowel, patients, *n* (%)	18 (60.0%)	12 (40.0%)	0.0721
Ileo-colonic or colonic, patients, *n* (%)	7 (100.0%)	0	
*UC*			
Proctitis, patients, *n* (%)	1 (14.3%)	6 (85.7%)	0.1189
Left side or extensive colitis, patients, *n* (%)	5 (62.5%)	3 (37.5%)	

GLIM = Global Leadership Initiative on Malnutrition; DASI = Duke Activity Status Index.

**Table 5:** Diagnosis of Malnutrition.

	IBD	CD	UC	p *
Malnutrition according to GLIM criteria, *n* (%)	22 (42%)	13 (34%)	9 (60%)	0.1236
Stage 1, *n* (%)	8 (15%)	6 (16%)	2 (13%)	0.6581
Stage 2, *n* (%)	14 (27%)	7 (18%)	7 (47%)	0.0724
Malnutrition according ESPEN 2015 criteria, *n* (%)	14 (27%)	7 (18%)	7 (47%)	0.0724
Concordance between the 2 criteria	K 0.672			

* *p*-values CD vs. UC.

**Table 6:** Prevalence of high nutritional risk according to different screening tools.

	IBD	CD	UC	p *
NRS-2002				
<3, *n* (%)	32 (60%)	26 (68%)	6 (40%)	0.0696
≥ 3, *n* (%)	21 (40%)	12 (32%)	9 (60%)	
MUST				
<2, *n* (%)	38 (72%)	31 (82%)	7 (47%)	**0.0179**
≥2, *n* (%)	15 (28%)	7 (18%)	8 (53%)	
MST				
<2, *n* (%)	38 (72%)	30 (79%)	8 (53%)	0.0915
≥2, *n* (%)	15 (28%)	8 (21%)	7 (47%)	
SaskIBD-NR				
<5, *n* (%)	40 (75%)	29 (76%)	11 (73%)	>0.999
≥5, *n* (%)	13 (25%)	9 (24%)	4 (27%)	
MIRT				
<3, *n* (%)	32 (60%)	26 (68%)	6 (40%)	0.0696
≥3, *n* (%)	21 (40%)	12 (32%)	9 (60%)	

* *p*-values CD vs. UC. NRS-2002 = Nutritional Risk Screening 2002; MUST = Malnutrition Universal Screening Tool; MST = Malnutrition Screening Tool; SaskIBD-NR = Saskatchewan IBD–Nutrition Risk; MIRT = Malnutrition Inflammation Risk Tool.

**Table 7:** Concordance of GLIM diagnosis of malnutrition with the prevalence of high nutritional risk according the screening tools used.

	NRS-2002	MUST	MST	SaskIBD-NR	MIRT
Sensitivity%	81.82	63.64	63.64	50	81.82
[95% CI]	[59.72% to 94.81%]	[40.66% to 82.8%]	[40.66% to 82.8%]	[28.22% to 71.78%]	[59.72% to 94.81%]
Specificity%	90.32	96.77	96.77	93.55	90.32
[95% CI]	[74.25% to 97.96%]	[83.3% to 99.92%]	[83.3% to 99.92%]	[78.58% to 99.21%]	[74.25% to 97.96%]
LR	8.455	19.73	19.73	7.75	8.455
Area under the ROC curve	0.9194	0.8783	0.8768	0.9032	0.7757
*p* value	<0.0001	<0.0001	<0.0001	<0.0001	<0.0007

NRS-2002 = Nutritional Risk Screening 2002; MUST = Malnutrition Universal Screening Tool; MST = Malnutrition Screening Tool; SaskIBD-NR = Saskatchewan IBD–Nutrition Risk; MIRT = Malnutrition Inflammation Risk Tool; LR = Likelihood Ratio; CI = Confidence Interval; ROC = Receiver Operating Characteristics.

## Abbreviations

IBD	Inflammatory bowel disease
CD	Crohn’s disease
UC	Ulcerative colitis
GLIM	Global Leadership Initiative on Malnutrition
BIVA	Bioelectrical Impedance Vector Analysis
CRP	C-Reactive Protein
WBC	White Blood Cells
GI	Gastrointestinal
UWL	Unintended Weight Loss
BMI	Body Mass Index
FFM	Free Fat Mass
FFMI	Free Fat Mass Index
NRS-2002	Nutritional Risk Screening 2002
MUST	Malnutrition Universal Screening Tool
MST	Malnutrition Screening Tool
MIRT	Malnutrition Inflammation Risk Tool
SaskIBD-NR	Saskatchewan IBD–Nutrition Risk
DASI	Duke Activity Status Index
